# Direct Utilization of Organic Nitrogen by Phytoplankton and Its Role in Nitrogen Cycling Within the Southern California Bight

**DOI:** 10.3389/fmicb.2018.02118

**Published:** 2018-09-13

**Authors:** Michael Morando, Douglas G. Capone

**Affiliations:** Marine and Environmental Biology, University of Southern California, Los Angeles, CA, United States

**Keywords:** stable isotope probing, DNA-SIP, function, microbial diversity, nitrogen cycling, new and regenerated production, urea assimilation, diatoms

## Abstract

The new production model attempts to quantify the amount of organic material exported from surface waters based on the form of nitrogen (N) being utilized. Dissolved organic N (DON) is rarely assessed during such investigations and even less is understood about the organisms involved in these different transformations within the complex N cycle. Stable isotope probing (SIP) and uptake activity measurements were combined to investigate the dynamics of new and regenerated production during the spring within the Southern California Bight (SCB). We examined the uptake and assimilation of several nitrogenous substrates at several depths to quantify these processes and identify the active communities across all three domains of life that are driving each transformation. Several reoccurring members closely related to the eukaryotic diatom *Chaetoceros*, dominated assimilation of NO_3_^-^ and urea through the water column, and contributed greatly to the overall production. Prokaryotic growth was predominantly carried out through NH_4_^+^ assimilation with transitions from Flavobacteria to *Rhodobacteraceae* and Marine Group II Euryarchaeota to Marine Group I Thaumarchaeota with increasing depth for bacterial and archaeal clades, respectively. Only urea uptake and SIP activity correlated with each other, likely demonstrating that cellular transport and incorporation of urea were coupled. SIP was therefore able to identify the organisms primarily responsible for urea cycling at each depth during this investigation. The role of diatoms within high nutrient areas are well defined but their part in DON cycling in highly stratified regimes is less well understood. Here we demonstrate their ability to efficiently scavenge urea *in situ*, allowing certain diatoms to outcompete the rest of the community. This diversion of DON away from the trophically inefficient microbial loop directly back into the larger, particle forming populations would alter the current view of microbial food webs. This proposed “phytoplankton shunt” of organic material could potentially enhance the biological pump by mitigating losses due to trophic transfers while increasing DON flux due to ballasting. Our results provide unique biogeochemical and ecological insight into the dynamics and diversity of N cycling and the organisms involved within the surface waters of the SCB.

## Introduction

The fate of marine primary production has been of ecological interest for decades due to its implications for the control of the biological pump and carbon sequestration. Constraining the factors that influence the magnitude and variability of production is critical to understanding marine ecosystems. Production can be further broken down into two main categories, that which relies on allochthonous Nitrogen (N) sources (imported or “new production") and that which is recycled within the system (autochthonous or “regenerated production"). According to the new production model (Dugdale and Goering, 1967; Eppley and Peterson, 1979), new production is generally associated with net import fluxes of NO_3_^-^, while recycled production depends largely on NH_4_^+^ released by heterotrophic consumption within the system. It was not until decades after this model’s inception that N_2_ fixation, a new N source, was fully considered. Together, they provide a basis to estimate total productivity as well as C export and export efficiency which can be readily compared among different systems (Eppley et al., 1979; Platt and Harrison, 1985; Harrison et al., 1987; Aufdenkampe et al., 2001).

Traditionally studies investigating these processes focus on dissolved inorganic N (DIN) sources, i.e., NH_4_^+^ and NO_3_^-^, but typically do not assess organic N even though it is the second most abundant form of fixed N in the ocean (Bronk, 2002). The degradation of organic matter is critical in turning over resources that become “trapped” in detritus and a portion of this is released as dissolved organic N (DON). Without this process, the euphotic zone would have to solely rely on new sources of N to fuel primary production, which can be slow and limited in certain environments, e.g., by the diffusive flux of NO_3_^-^ from below the nitracline (Falkowski, 1998).

Organic N, primarily in the form of urea, has been shown to be an important N source in aquatic systems, especially when NO_3_^-^ concentrations are low, usually ranging from 15 to 80% of total N uptake (Varela and Harrison, 1999; Bronk, 2002; Bradley et al., 2010), averaging ∼20% across a diverse range of marine systems (Bronk, 2002; Mulholland and Lomas, 2008). Despite this, urea is often missing from biogeochemical and ecological surveys (Antia et al., 1980), with NH_4_^+^ representing the only measured regenerated source (Eppley and Peterson, 1979; Harrison et al., 1987). Rates of urea uptake are not well characterized globally but are often comparable to and can exceed that of NH_4_^+^; ignoring this aspect of production in N uptake surveys was shown to grossly underestimate primary production over a variety of ecosystems (Wafar et al., 1995). *f*-ratios estimated from these surveys, used to assess the proportion of production derived from new N sources, were inflated by as much as 55% compared to calculations including urea uptake (Wafar et al., 1995).

Further complicating our understanding of these dynamics is the lack of information on the identities of individual organisms responsible for the mediation of these processes *in situ*, such as organic matter degradation, as well as the mechanisms that regulate them (Berman and Bronk, 2003). Most new production investigations rely almost exclusively on biogeochemical measurements, with the composition of the community involved being viewed as a “black box” (Aufdenkampe et al., 2001; Michaels et al., 2001; Lipschultz et al., 2002). This was done originally to avoid further complicating nutrient-phytoplankton-zooplankton (NPZ) models due to limited computing capacity, necessitating the use of a simplistic view of new and regenerated production, where distinct roles for phytoplankton, i.e., new N uptake, and the prokaryotic, i.e., recycling of organic matter, communities were pre-defined. We now know that N cycling is much more dynamic with members from all three domains of life participating in a multitude of facets at each level of these transformations (Zehr and Ward, 2002; Zehr and Kudela, 2011). Yet, questions still remain about the diversity of these organisms as well as the factors that regulate their individual activity.

Stable isotope probing (SIP) was employed to provide a more complete view of new versus regenerated production by combining this relatively new ecological technique with more traditional biogeochemical methods. SIP is able to provide a unique perspective into nutrient cycling because the identity of the organisms actively involved in the transformation of specific ^15^N-substrates is directly determined through the incorporation of the isotopically labeled N into their DNA (Cadisch et al., 2005; Buckley et al., 2008; Wawrik et al., 2009; Orsi et al., 2018). SIP’s power comes from its ability to identify active assimilators of these substrates in the environment, particularly uncultivated taxa (Buckley et al., 2007b; Nelson and Carlson, 2012; Orsi et al., 2016; Morando and Capone, 2016) where often little, if anything is known about their ecological roles or metabolic functions. Multiple N substrates were implemented to assess both bulk uptake rates as well as the composition of the active prokaryotic and eukaryotic communities using SIP. Our goal was to identify the major players involved in new and regenerated production within the photic zone of the Southern California Bight (SCB) and thereby to better understand the dynamics of their marine microbial food webs. The distribution of this activity was also assessed over several depths to further understand how differences in environmental parameters, e.g., nutrients and light, may have influenced these community compositions. To the best of our knowledge, the employment of multiple substrates over multiple depths has not been attempted previously.

## Materials and Methods

### Water Sampling

Water was collected on the morning of April 10, 2014 aboard the R/V Yellowfin during a monthly cruise at the San Pedro Ocean Time-series (SPOT; 33°33′N, 118°24′W). SPOT is located ∼20 km offshore within the SCB and has a depth of ∼900 m. A Niskin rosette system was used to collect water from surface, 10% light, and 1% light, as determined by PAR sensor measurements taken during the retrieval of the samples. These light levels corresponded to depths of approximately 5, 17, and 35 m, respectively (**Table 1**). All samples were stored in coolers on blue ice until it was transported back to our laboratory at the University of Southern California for further processing.

**Table 1:** Characteristics of the sampling site.

			Ambient			SIP amendments		
Percent light	Depth	[Chl a]	[NH_4_^+^]	[NO_3_^-^]	[Urea]	[NH_4_^+^]	[NO_3_^-^]	[Urea]
	m	μg l^-1^	μM	μM	μM	μM	μM	μM
50	5	0.31 (0.01)	0.20 (0.06)	0.10 (0.02)	0.28 (0.03)	0.8	1.0	1.0
10	17	0.54 (0.02)	0.25 (0.05)	0.15 (0.02)	0.32 (0.005)	0.8	1.0	1.0
1	35	2.75 (0.05)	0.76 (0.03)	10.7 (0.1)	0.75 (0.01)	0.8	20.0	1.0


*Brackets designate concentrations measured with the values in parentheses representing the standard deviation of the mean.*

### Environmental Variables

Hydrographic data was collected from depth using a CTD system. Water collected from each depth was either processed within 4–8 h or frozen at -20°C for later analysis. Concentrations of NH_4_^+^ and urea were measured (triplicate) as previously described (Price and Harrison, 1987; Holmes et al., 1999; Taylor et al., 2007). NO_x_ concentrations (the combined measurement of NO_3_^-^ plus NO_2_^-^ and will be referred to as NO_3_^-^ from here on) were analyzed (in triplicate) at the Marine Science Institute Analytical Laboratory at University of California, Santa Barbara by standard colorimetric methods (Parsons et al., 1984). Chlorophyll samples (triplicate) were processed as previously described (Holm-Hansen and Riemann, 1978).

### ^15^N-Isotope Experiments

All isotope experiments were done in acid-washed polyethylene bottles, followed by three rinses with ambient seawater. Quadruplicate 1 L samples from each depth were amended to a final concentration of 0.03, 0.03, and 0.015 μM, with ^15^N-labeled (> 98% ^15^N) NO_3_^-^, urea, or NH_4_^+^ (Sigma-Aldrich, St. Louis, MO, United States), respectively, to assess the uptake rates of the microbial community. Three ml of ^15^N_2_ gas (Sigma-Aldrich, St. Louis, MO, United States) were used to measure N fixation in 2 L incubations. A single bottle was filtered immediately to establish a T_0_ atom% ^15^N of the particulate N for each substrate at each depth. The remaining bottles were placed in circulating temperature-controlled incubators that were shaded by different mesh size combinations of aluminet screening to simulate ambient light intensity and temperature of the collection site. Incubations were carried out for ∼24 h. All samples were filtered onto precombusted (∼5 h at 400°C) 25 mm GF/F filters (Whatman, Maidstone, VT, United States), dried, and stored until analysis on an IsoPrime continuous flow isotope ratio mass spectrometer (CF-IRMS). IRMS data were corrected for both size effect and drift before being calculated as previously described (Dugdale and Goering, 1967).

Stable isotope probing samples were amended with ^15^N-labeled (experimental sample) or ^14^N-unlabeled (control sample) NH_4_^+^, NO_3_^-^, or urea. Samples were incubated under *in situ* light and temperature as described above. Incubations were terminated after ∼24 h by peristaltic filtration onto 0.2 μm Supor filters (Pall Life Sciences, Ann Arbor, MI, United States), immediately flash frozen, and stored under -80°C until extraction in the laboratory. Final concentrations for all SIP amended samples can be found in **Table 1**.

### DNA Extraction and CsCl Gradient Ultracentrifugation

DNA was extracted from SIP samples using DNeasy kit (Qiagen, Hilden, Germany) with additional bead beating (30 s) prior to extraction. DNA was quantified with the Qubit assay (Invitrogen, Carlsbad, CA, United States) and ∼2000 ng of DNA from each sample was added to separate centrifuge tubes containing CsCl. Centrifugation was carried out in an NVT65.2 rotor (Beckman Coulter, Indianapolis, IN, United States) at ∼44 krpm for ∼66 h at 20°C. Gradients were fractioned intact and purified based on a modified protocol (Neufeld et al., 2007). Individual gradients were displaced by mineral oil from above and collected from below as fifty ∼100 μl fractions. Each fraction’s density was determined using a modified AR200 handheld digital refractometer (Reichert, United States), as described by Buckley et al. (2007a). DNA was purified by the addition of two volumes 30% polyethylene glycol (PEG) solution and 20 μg glycogen with a final 70% ethanol wash.

After elution in 30 μl of TE buffer, the distribution of DNA in the CsCl gradient was determined through the quantification of each fraction using the Qubit assay (Invitrogen). Thirty fractions containing almost all of the bulk DNA distribution from both isotopically labeled and control samples were selected for further processing from each experiment. PCR was carried out on each of these fractions using fusion primers that amplified a segment of the V4-V5 region of the 16S rRNA gene (515F 5′-GTGYCAGCMGCCGCGG) and 926R (5′-CCGYCAATTYMTTTRAGTTT), which allowed multiplexing through the use of an inline five base pair (bp) barcode on the forward primer and a unique six bp index on the reverse primer (Huse et al., 2014). PCR conditions and sequences of taxa potentially not amplified by these primers are discussed extensively in Parada et al. (2015). All amplicons were sequenced on the Illumina MiSeq platform at the University of California, Davis sequencing core. These reads were first quality filtered using Trimmomatic (Bolger et al., 2014), prior to merging forward and reverse reads. Mothur (Schloss et al., 2009) was then used to stich these reads together and further quality filter Chimeras were removed using UCHIME (Edgar et al., 2011) and clustered into OTUs (at 99%) using the average neighbor algorithm in Mothur (Schloss et al., 2009). Taxonomy was assigned with Mothur (Schloss et al., 2009) using the SILVA database v123 (Quast et al., 2013) available on the Mothur wiki at the time this data was analyzed. OTUs with low abundance over the majority of fractions being assessed at each depth were removed from further analysis to minimize potential artifacts that may be caused by random banding of low abundance organisms. Chloroplast sequences analysis followed the methodology outlined and established by Needham and Fuhrman (2016). The analysis of dinoflagellate chloroplasts were not part of this study due to the abnormal nature of these sequences (Koumandou et al., 2004), so this fraction of the eukaryotic community has not been evaluated in these results. Genomic data have been deposited in the European Nucleotide Archive under accession numbers ERS2402332 to ERS2402751.

An R script was created to process sequencing and gradient data, isolating and evaluating the DNA bands of each OTU within their respective gradients. The identity of density shifts, and therefore assimilation, of the labeled substrate for each taxon was then determined. Only DNA bands of the same OTU were compared to limit organism-to-organism variations, such as GC content, which has been shown to affect DNA density (Buckley et al., 2007a). Read counts were converted to relative abundance within each fraction and low abundance fractions were removed. The bulk DNA distribution measured within each gradient was used to convert relative abundance into ng DNA per fraction for each specific OTU, and finally to the percentage of DNA for each OTU per fraction. Tails of these distributions were trimmed to the same approximate densities and reintegrated to better isolate each DNA band and facilitate comparisons. DNA density was determined for each individual OTU by calculating the weighted mean density of the entire DNA band measured over all fractions sequenced. Weighted mean densities were compared between individual controls and treated OTUs to determine if isotopic incorporation of the labeled substrate occurred.

The incorporation of unlabeled N does not affect the buoyant density of DNA. Any variation in banding between the controls of an individual OTU, i.e., incubated with unlabeled N substrates, is likely due to the measurement precision associated with that particular OTU (Hungate et al., 2015). Therefore, all control fractions from a single OTU were combined over all treatments prior to calculating DNA density (**Supplementary Figure 1**). This enabled a better estimation of the mean density and variance for the native unlabeled DNA of each individual control OTU, crucial to evaluating changes in density concomitant with isotopic uptake. Pooled control DNA density was then compared to a weighted mean and variance estimate for each OTU exposed to isotopically labeled substrate, similarly to Hungate et al. (2015). Statistically significant shifts in DNA density were evaluated using Welch’s *t-*test, which can control for potential differences in variances and samples sizes.

Fractions from OTUs with low total reads can greatly affect the precision and accuracy of determining the overall density of each OTU’s DNA band, since the presences or absence of just a few reads could drastically affect the calculated mean density (Connelly et al., 2014). OTUs with fractions containing such low abundance were removed prior to *P-value* corrections for multiple comparisons using the Benjamini-Hochberg method (Benajmini and Hochberg, 1995), with a false discovery rate of 0.1 [a typical threshold used in molecular analysis, including previous SIP work (Pepe-Ranney et al., 2016)]. Shifts in DNA density identified through this analysis (**Supplementary Figure 1**) represent the incorporation of ^15^N and ultimately activity associated with the particular substrate being evaluated in each experiment.

Stable isotope probing activity at the OTU level and bulk uptake of each N source was measured in parallel. Uptake activity occurs upon the transport of substrate into the cell and while no utilization of the substrate is required, it is often assumed. This may hold true generally, but instances of uptake and incorporation becoming decoupled do occur (Falkowski and Raven, 1997; Falkowski et al., 1998) and the conditions that promote this are likely different among and within phylogenetic clades and even individual cells. SIP activity goes a step further than uptake, by also requiring assimilation of the substrate into DNA, providing insight into the potential role individual organisms play in the both the transport and utilization of specific N sources.

Each OTU has to reach a certain threshold of relative abundance and ^15^N incorporation in order to be identified as enriched, with previous N based SIP studies requiring > 30% DNA enrichment for positive identification of assimilation (Murrell and Whiteley, 2011; Connelly et al., 2014). This in combination with short incubation periods, i.e., 24 h, facilities the targeting of the most active fraction of the microbial community that are contributing to the bulk of N assimilation. Isotopically enriched OTUs identified in this study generally had similar shifts in DNA density, suggesting comparable rates of assimilation. Though the absolute amount of N assimilation for each substrate could not be quantified per OTU, the total number of OTUs actively assimilating each N substrate was used to estimate the potential amount of N assimilation occurring at each depth, assuming each OTU present had a similar assimilation potential. Assimilation potential would therefore increase as the number of active organisms identified via SIP increased. This framework was employed in an attempt to compare bulk uptake rates and organismal level assimilation to identify common trends between these related but independent measures of metabolism.

A rotor failure occurred during an ultracentrifugation causing the loss of the 10% light NO_3_^-^ set of SIP samples and so no data was generated or included in any of the analysis.

### Phylogenetic Analysis

16S rRNA gene sequences of SIP OTUs of interest were aligned in Geneious (Kearse et al., 2012) with related publically available sequences obtained from GenBank (Benson et al., 2015) with ClustalW (Thompson et al., 2002). The K80 model (Kimura, 1980) was used to construct a maximum-likelihood tree with PhyML (Guindon et al., 2010), which were bootstrapped 1000 times.

## Results

### Water Sampling and Environmental Variables

**FIGURE 1:**
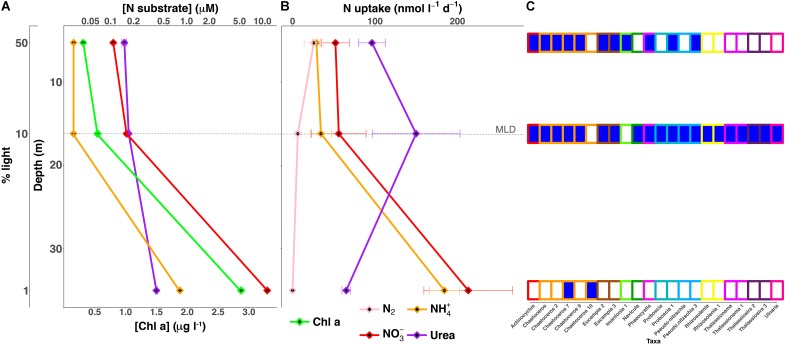
Depth profiles of nutrient and uptake rates as compared to eukaryotic OTUs driving urea assimilation. **(A)** NO_3_^-^ (red), NH_4_^+^ (orange), urea (purple), and Chl a (green) concentrations **(B)** and uptake rates measured at each light level; N-fixation was also measured (pink). **(C)** SIP activity for the eukaryotic OTUs controlling urea assimilation found at each sampling depth depicted in the nutrient and uptake profiles **(A,B)**. Urea utilizers, OTUs showing DNA enrichment, are identified by a dark blue square and the outline color of each box corresponds to a specific clade. Each measurement corresponds to a specific light level that is designated on the far left axis. Dashed line represents the mixed layer depth (MLD).

The water column was highly stratified with a shallow mixed layer depth (MLD) that was estimated to be ∼17 m (**Figure 1A**), the depth where σ_𝜃_ differed by 0.125 kg m^-3^ from the surface. The upper mixed layer where the two shallower samples were collected was nutrient-poor (**Table 1** and **Figure 1A**). A third sample was collected from the 1% light level layer in the well-mixed nutrient-rich waters below (**Table 1** and **Figure 1A**). NH_4_^+^ and NO_3_^-^, were quite low above the MLD, while concentration maxima for both nutrients were found at the 1% light level (**Table 1** and **Figure 1A**). Urea concentrations were relatively higher than dissolved inorganic nitrogen (DIN) within the mixed layer and also peaked at the deepest depth. The deep chlorophyll maxima (DCM) coincided with the 1% light level at 35 m, with 2.75 μg l^-1^, and was the second highest measured chlorophyll concentration at this or any depth during 2014 at SPOT.

### ^15^N-Isotope Experiments

Bulk uptake rate experiments were carried out over a 24 h period in order to capture both day and night periods and were treated as net rates integrated over a full day. DIN uptake rates within the upper mixed layer were fairly constant with an increase of ∼4-fold at the 1% light level, where it comprised ∼86% of the total uptake (**Figure 1** and **Supplementary Table 1**). A sharp transition occurred in the major N source from a predominance of urea uptake in the upper mixed layer where urea accounted for ∼55% of total integrated uptake, with a peak at the 10% light level, dropping to only ∼14% at the 1% light level. N_2_ fixation was measureable but low above the MLD, and decreased to below detection at the DIN rich 1% light level (**Figure 1A** and **Supplementary Table 1**). Regenerated N sources, NH_4_^+^ and urea, fueled production above the 1% light level, where new production, NO_3_^-^ uptake and N fixation, comprised less than a third of total production. In contrast, below the MLD, regenerated production was just slightly below 40%. Total integrated nitrogen uptake for this sampling site was ∼9 mmol N m^-2^ d^-1^, sufficient to support ∼60 mmol C m^-2^ d^-1^ (**Table 2**) based on a Redfield conversion (Redfield, 1963). Urea uptake accounted for ∼38% of total integrated N uptake and was the major source of nitrogen supporting production during this sampling, followed closely by nitrate uptake. An *f*-ratio, calculated as the ratio of new (the sum of NO_3_^-^ uptake and N fixation) to total production (the sum of N fixation, NH_4_^+^, urea, and NO_3_^-^ uptake; (Eppley and Peterson, 1979), of ∼0.37 was derived from the total integrated production (**Table 2**).

**Table 2:** Total N uptake rates integrated over the entire euphotic zone.

Substrate	N uptake	Total uptake^a^	C fixation^b^
	mmol N m^-2^ d^-1^	Fraction	mmol C m^-2^ d^-1^
Urea	3.4	0.38	21.1
NO_3_^-^	3.1	0.34	20.6
NH_4_^+^	2.4	0.26	15.8
N_2_	0.19	0.02	1.3
Total	8.8	1.0	58.7


*^a^f-ratio was equal to ∼0.37.*

*^b^Converted from N uptake using C:N 6.7.*

### Identification of Activity by ^15^N Incorporation by Individual OTUs

**FIGURE 2:**
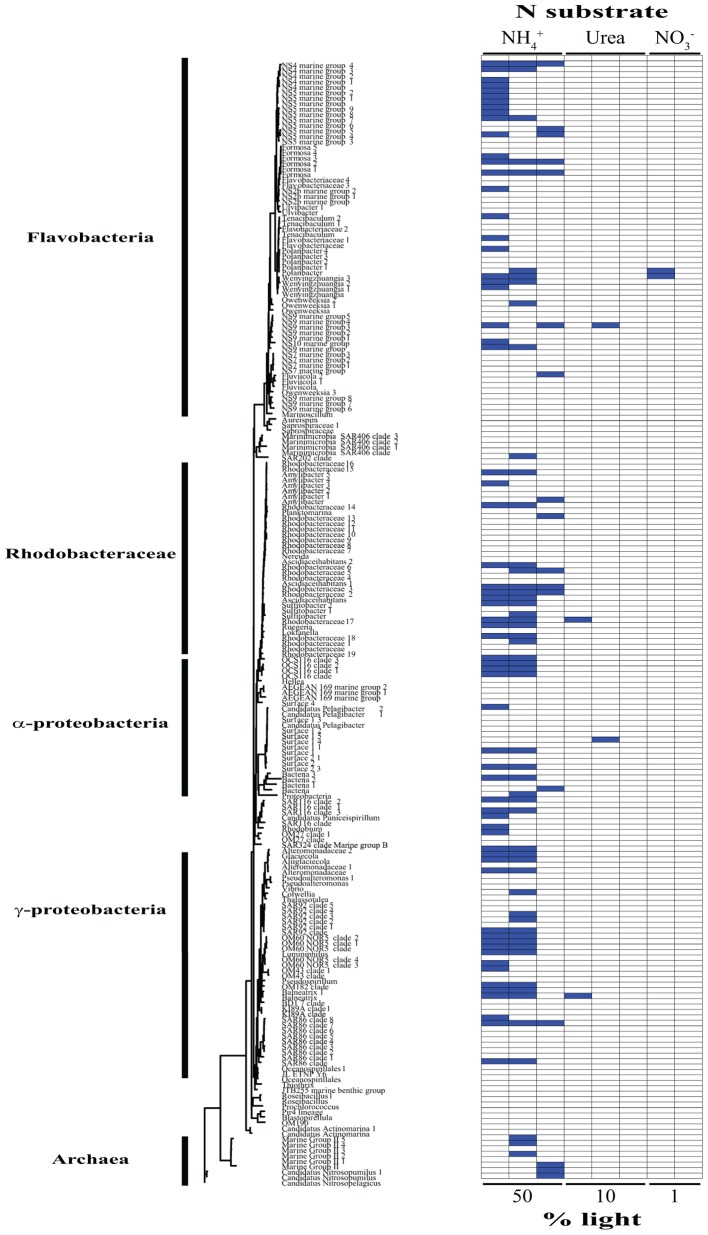
Phylogenetic tree of all prokaryotic OTUs assessed during this study illustrating which taxa are actively assimilating each substrate at each light levels. Active utilizers, OTUs showing DNA enrichment of a specific N substrate, are identified by a dark blue square. Phylogenetic clades of interested have been designated of left.

**FIGURE 3:**
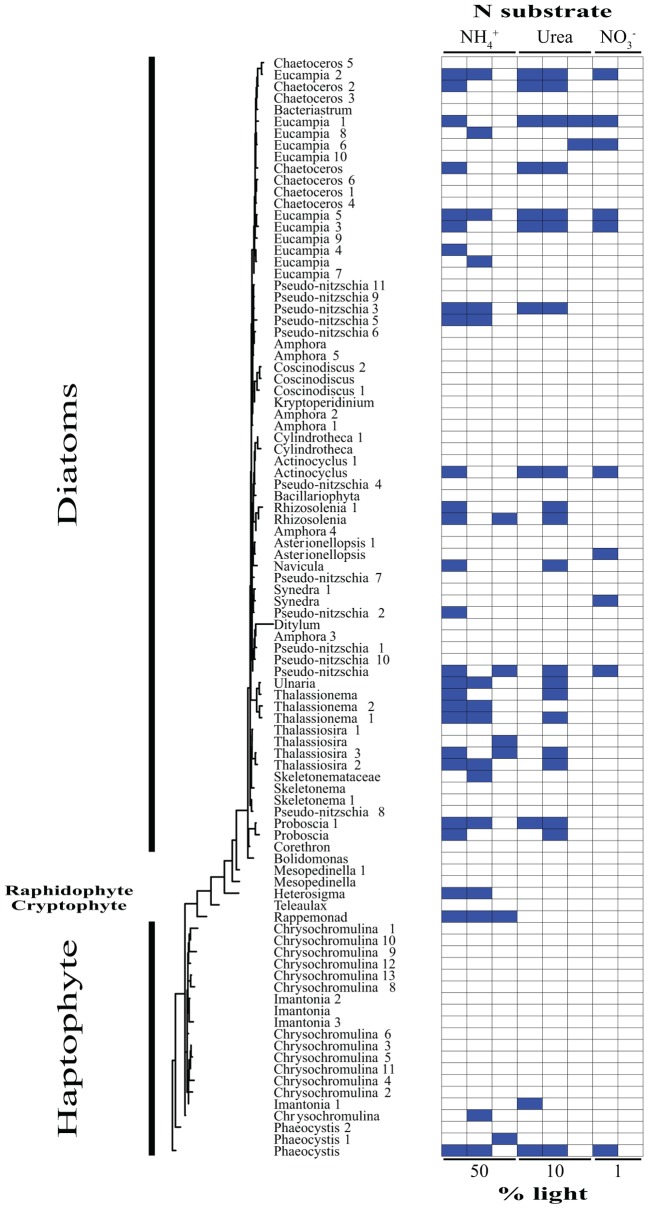
Phylogenetic tree of all eukaryotic OTUs assessed during this study illustrating which taxa are actively assimilating each substrate at each light levels. Active utilizers, OTUs showing DNA enrichment of a specific N substrate, are identified by a dark blue square. Phylogenetic clades of interested have been designated of left.

Based on our SIP analysis, the prokaryotic fraction of active assimilators dominated uptake over all light levels (**Supplementary Figure 2**) and was composed almost entirely of two phyla, Bacteroidetes and Proteobacteria, each making up ∼50% of the total (**Figure 2**). Enrichment was largely restricted to NH_4_^+^ assimilation, ∼96% of enriched prokaryotes were found within these treatments. Only six prokaryotic OTUs were isotopically labeled with either NO_3_^-^ or urea across all incubations, accounting for ∼12% of the combined activity within these treatments (**Figure 2**), while prokaryotes in general composed ∼74% of the active community across all NH_4_^+^ treatments (**Figure 2** and **Supplementary Figure 3**). The eukaryotic phototrophic fraction, as determined by chloroplast identity (see section “Materials and Methods”), encompassed ∼12% of the total active community across all incubations, representing ∼38% (36 OTUs) of the eukaryotic phototrophic community assessed (**Figure 3**). OTUs with high sequence-similarity to diatom chloroplasts, including multiple members of *Chaetoceros* spp. and *Pseudo-nitzschia* *seriata*, largely represented the active eukaryotic community (**Figure 3**).

The type of N substrate assessed in each incubation appeared to have a greater effect on SIP activity than light. The majority of enriched OTUs were found in the NH_4_^+^ treatments (∼79%) (**Supplementary Figure 3** and **Figures 2**, **3**), which greatly influenced the overall SIP results. Light did, however, affect the number of OTUs enriched, as the total amount of SIP activity decreased with decreasing light (**Supplementary Figure 2**). The biggest decline occurred from the 10% to the 1% light level, where < 3% of all OTUs examined were enriched. Of the enriched OTUs from the 1% light level, none were enriched with NO_3_^-^ and only two showed significant enrichment from urea, accounting for just ∼18% of the active OTUs at this depth. Both urea-enriched OTUs were identified as photosynthetic eukaryotes. Urea was the only substrate to deviate from this trend of diminishing SIP activity with depth and had a slight increase in the total number of enriched OTUs at the 10% light level before falling to close to zero at the 1% light level. Light appeared to have less of an effect on the overall SIP activity of the prokaryotic community relative to the eukaryotic community. The ratio of enriched prokaryotes to photosynthetic eukaryotes remained fairly constant above the MLD, however, an increase was observed at the 1% light level, where eukaryotes only composed ∼32% of the enriched community (**Supplementary Figure 2**).

Enrichment of each OTU was compared over all treatments to identify each organism’s relative contribution to SIP activity. OTUs were considered to be more active as the number of treatments in which they were isotopically labeled increased, since this would indicate that they were either able to assimilate multiple substrates and/or show activity at multiple depths. These organisms likely made a relatively greater overall contribution to activity and N cycling at SPOT during the time of this study. OTUs enriched in at least two or more treatments made up ∼24% of the total population assessed. Of these enriched OTUs, ∼35% were photosynthetic eukaryotes, an increase of almost 3× compared to the eukaryotic fraction of OTUs labeled in at least one treatment. *Phaeocystis* *globosa* and several diatom clades were active and assimilating in five of the eight treatments. The prokaryotic community showed greater diversity in activity, where enrichment was spread out over a higher percentage of organisms, with no single OTU being enriched in more than three different treatments. On average, an enriched prokaryotic OTU was shown to be statistically significantly enriched, via Welch’s *t*-test, in 1.6 treatments, vs. 2.5 for a eukaryotic taxon. Several members of the *Flavobacteriaceae* and *Rhodobacteraceae* were among the most active OTUs of the prokaryotic community.

## Discussion

### N Uptake and SIP Activity

The increased availability of nutrients with depth likely drove the increase in overall uptake (**Figures 1A,B**). However, a similar trend was not observed in SIP activity, as defined by the total number of active OTUs, which decreased over this same range (**Supplementary Figures 2, 3**). This may point toward nutrient concentrations potentially having a stronger effect upon transport and uptake with light playing a larger role in SIP activity due to the great costs associated with assimilation. This was bolstered by enriched OTU’s minimums occurring for each substrate at the lowest light level (**Supplementary Figure 3**), with enrichment minimums for both NH_4_^+^ and NO_3_^-^ occurring at depth despite peak DIN concentrations and chlorophyll (**Figures 1A,B**). Less light and, therefore, less energy can lead to reduced growth efficiency and rates (Hickman et al., 2009, 2012), which may have influenced the increased disassociation seen between the uptake and assimilation potential with depth.

Storage of substrate prior to incorporation could also affect SIP activity with many of the taxa present during this study, e.g., *Chaetoceros*, *Thalassiosira*, and *Skeletonema*, having the capacity to take up and store nutrients (Dortch, 1982; Dortch et al., 1985). Internal storage without utilization would be quantified as uptake, but since SIP activity requires assimilation as well, no detectable DNA enrichment would occur. Even short periods of storage could still delay incorporation long enough to prevent significant amounts of DNA enrichment from taking place during these incubations.

Previous investigations of N transformations in the SCB have also found that a portion of N uptake is often unaccounted for in the particulate fraction and have linked this “missing DIN uptake” to DON release (Ward et al., 1989; Bronk and Ward, 2005). They reported that DON release increased dramatically within the nitracline relative to the nutrient poor surface and was generally higher with NO_3_^-^ compared to NH_4_^+^ as the nutrient source. Stukel et al. (2011) examined grazing just north of the SCB and found that phytoplankton growth rates exceeded grazing rates near the surface, but decreased rapidly with depth relative to grazing. This higher turnover at depth could result in the decoupling of uptake from utilization and incorporation by not allowing enough time for cellularly transported nutrients to be assimilated through multistep incorporation pathways.

These factors could help provide a possible explanation for the biggest discrepancies observed between uptake and assimilation in this study, which occurred at the lowest light level and included no detectable NO_3_^-^ SIP enrichment even though NO_3_^-^ uptake rates peaked (**Figure 1A** and **Supplementary Figures 2, 4**). Similar results were seen in the Alaskan Artic, where the incorporation of ^15^NO_3_^-^ by the community was not detected by SIP despite measurable rates of NO_3_^-^ uptake (Connelly et al., 2014). This missing NO_3_^-^ enrichment of the microbial community may be indicative of a large portion of new production not actually being incorporated into particles, lowering the probably of sinking and export.

In contrast to what was observed with the other substrates, urea SIP activity (**Supplementary Figure 3**) and diversity (**Figures 1C, 3**) actually increased at the 10% light level for eukaryotes, despite the decrease in availability of light energy. These increases coincided with an overall rise in urea uptake (**Figures 1B,C**), as well as the potential for eukaryotic urea utilization (**Supplementary Figure 3**), marked by a major increase in diatom enrichment and diversity (**Figures 1C, 3**), doubling the number of active organisms, including multiple OTUs of *Rhizosolenia*, *Thalassionema*, and *Thalassiosira* (**Figures 1C, 3**). The positive correlation between urea uptake and incorporation potential (**Figures 1B,C** and **Supplementary Figure 3**) may indicate that these two processes were more closely coupled than the other substrates investigated. SIP was therefore likely able to delineate the organisms responsible for the bulk of urea cycling during this study (**Figures 1B,C**), particularly those responsible for the increased uptake at the 10% light level (**Figures 1B,C**). Loss of activity by these taxa at the 1% light level (**Figure 1C**) may have played a role in the significant decrease in urea uptake below the MLD (**Figure 1B**). This strongly suggests that these clades affected this peak in urea uptake (**Figures 1B,C**), contributing a major portion of the 1.9 mmol N m^-2^ d^-1^ of regenerated production taking place in the mixed layer, 80% of which is associated with urea.

### Urea Assimilation by Diatoms

Profiles of nutrients, density, and temperature all demonstrated that these waters were highly stratified during the time of sampling (**Figure 1A** and CTD profiles not shown). This strong stratification likely acted as a barrier between the upper surface layer, i.e., 50 and 10% light levels, and the bottom of the euphotic zone, i.e., 1% light level, where DIN concentrations were elevated. Over time, this barrier would restrict DIN flux from depth to the surface, eventually increasing primary production’s reliance on the recycling of organic matter if stratification was not broken down. Similar conditions in previous studies have demonstrated urea’s potential to fuel regenerative production when other sources are low, on some occasions even stimulating, enhancing, and/or prolonging phytoplankton blooms (Kristiansen, 1983; Kudela and Cochlan, 2000; Glibert et al., 2001, 2006). Urea, a low-molecular weight, labile form of organic N, was the major N source fueling uptake within the mixed layer, eclipsing both DIN sources combined (**Figure 1A** and **Supplementary Table 1**). Eukaryotic phytoplankton dominated urea assimilation, which was highest within the mixed layer, increasing by almost twofold at its base (**Figures 1B,C, 3**). Phytoplankton also dominated urea uptake within the relatively organic-rich surface waters of the Mid-Atlantic Bight (Bradley et al., 2010).

The main utilizers of urea within the mixed layer were broadly identified as diatoms (**Figures 1C, 3**). The most active clade across all depths was primarily composed of OTUs most closely related to several genera of large, centric diatoms, i.e., several *Chaetoceros* spp. (**Figure 3**). These diatoms were assimilating urea at both depths above the mixed layer, comprising ∼45% of the urea assimilating fraction at the uppermost light level, declining to below 30% at the base of the mixed layer (**Figure 3**). Their N demand was satisfied concurrently by both NH_4_^+^ and urea at the 50% light level, suggested by the enrichment of the same OTUs in each treatment (**Figure 3**). This multi-substrate growth for these organisms continued at the 10% light level, where it peaked (**Figure 3**) along with rates of urea uptake (**Figure 1B**). Multi-substrate utilization by organisms broadly identified as diatoms, i.e., NH_4_^+^, NO_3_^-^, and urea assimilation, was shown via SIP in the surface waters of the west Florida shelf (Wawrik et al., 2009). The only activity found below the mixed layer at SPOT amongst these diatoms were from members of the *Chaetoceros* spp. and these organisms were solely utilizing urea (**Figures 1C, 3**).

Previous culture work on diatoms has uncovered a functional ornithine urea cycle along with evidence of near-constitutive protein turnover and production of urease regardless of N substrate or availability (Allen et al., 2011). This may be indicative of having the capacity to rapidly take up, breakdown, and utilize urea to fuel anaplerotic reactions, promoting the generation of intermediates for critical metabolic pathways, e.g., glutamine synthetase/glutamate synthase cycle, proline syntheses, and tricarboxylic acid cycle (Allen et al., 2011). Diatoms dominated urea assimilation, composing ∼69, ∼86, and 100% of the enriched OTUs within each incubation moving from the surface to depth (**Figures 1C, 3**). The agreement between bulk uptake and assimilation of this substrate within the mixed layer (**Figures 1B,C**) and the relatively short incubations lengths, i.e., 24 h, implies these organisms are not only likely driving the uptake of urea, but also its incorporation into biomass (**Figures 1B,C**). This apparently quick and efficient utilization and incorporation of urea provides evidence for their use of an active urea cycle *in situ*. Allen et al. (2011) hypothesized factors such as these likely facilitate growth, contributing to their ability to outcompete other members of the community. Though this was not evaluated directly in the current study, our findings support this hypothesis.

Diatoms’ ability to take advantage of organic N release may provide further explanation for their widespread distribution and major contribution to production, including in stratified N-poor surface waters. By utilizing DOM directly, diatoms may help form a “phytoplankton shunt” that circumvents the heterotrophic microbial loop that loses a relatively large portion of C to respiration. Redirecting DOM released by phytoplankton back into the larger size fractions of the marine food web could influence the biological pump. The role this plays in DOM cycling could have implications on future restructuring of global marine food webs as many climate change models predict an increase in stratification and subsequent drawdown of DIN in surface waters over time (Sarmiento et al., 1998; Falkowski and Oliver, 2007; Moore et al., 2018). Further work is required to fully understand the mechanism(s) controlling urea assimilation by diatoms and its distribution in environmental samples.

### Prokaryotic Influence on NH_4_^+^ Utilization

NH_4_^+^ assimilation was dominated by prokaryotes over all light levels (**Supplementary Figure 3**), with activity staying relatively constant moving from the surface to depth (**Figure 2** and **Supplementary Figure 3**). Flavobacteria, mainly members of clades NS2b, NS4, NS5, and NS9, decreased in activity at the 10% light level (**Figure 2**). This was accompanied by a rise in growth within the *Rhodobacteraceae*, which was the most active prokaryotic clade within the mixed layer (**Figure 2**). Only two of the active *Rhodobacteraceae* OTUs remained enriched at the 1% light level, with two new OTUs displaying metabolic activity (**Figure 2**). Here, *Bacteriodetes* represented the bulk of activity, caused mainly by an increase in *Flavobacteriaceae* (**Figure 2**).

Archaea activity was restricted to the two deeper depths, with NH_4_^+^ as the only N source fueling growth during this study (**Figure 2**). Several Marine Group II (MG-II) Euryarchaeota OTUs accounted for the archaeal activity above the MLD, while below there was a complete shift in the active community, switching to the Marine Group I (MGI) Thaumarchaeota *Nitrosopumilus* and *Nitrosopelagicus* (**Figure 2**). *Nitrosopumilus* and *Nitrosopelagicus* are both known as major contributors to the oxidation of ammonia to nitrite, the first step in nitrification (Könneke et al., 2005; Martens-Habbena et al., 2009; Santoro et al., 2010; Santoro and Casciotti, 2011), a key process regulating the global ocean reservoir of inorganic N (Ingalls et al., 2006). Their lack of activity above the 1% light level may be due to the light inhibition often associated with nitrification (Olson, 1981), together with the low availability of NH_4_^+^, allowing other organisms, such as MG-II, to outcompete them. Below the MLD, light inhibition may have been sufficiently relieved, facilitating the simultaneous use of NH_4_^+^ for both energy production and growth by these Thaumarchaeota, leading to a shift in the dominant archaeal taxa. Archaea had their greatest potential contribution to N assimilation below the MLD, comprising almost 20% of the active community as compared to 0 and ∼6% at the 50 and 10% light levels, respectively. This further demonstrates the importance of archaea in biogeochemical cycling at the base of the euphotic zone (Francis et al., 2005).

## Conclusion

The combination of uptake and SIP employed in this current study provides new insight into productivity within the SCB, including the identification of key organisms, their N substrate preferences, and their potential contribution to new and regenerated production. The lack of NO_3_^-^ SIP enrichment at depth brings into question what fraction of new N is being incorporated into biomass and eventually exported, while organic N is supporting a significant portion of production and metabolism with a strong correlation between transport (uptake rates) and assimilation (SIP enrichment). A diverse array of diatoms, led by a clade of several closely related, highly active *Chaetoceros* spp., drove the majority production, using both inorganic and organic N sources for growth.

Diatoms are known for their significant contribution to new production under nutrient rich conditions, forming the basis of some of the world’s most productive food webs, yet much less is understood regarding their role in organic N cycling. Here we reported several major clades that appear to be able to quickly scavenge organic material and efficiently utilize this for growth (**Figures 1C, 3**), i.e., within 24 h. Diatoms comprised at least 70% of the enriched urea SIP OTUs at each depth, suggesting they may also be able to outcompete other members of the community within stratified, nutrient poor surface waters. If much of the released organic material is incorporated directly back into phytoplankton, particularly into large diatoms with the potential to form long chains and aggregations, DOM’s fate may be altered from the canonical view (Azam and Malfatti, 2007). We propose a “phytoplankton shunt” within the marine microbial food web that could divert a portion of DOM away from the inefficient microbial loop, where organisms have relatively low sinking rates and high metabolic C demands (Azam and Malfatti, 2007). This shunt of organic matter directly back into these larger, particle forming populations, would further mitigate losses caused by trophic transfer while increasing DOM flux due to ballasting (Azam et al., 1983). Ultimately, this reintroduction of DOM directly into the phytoplankton community will increase the efficiency of the biological pump and the flux of N and C, additionally blurring the lines between new and regenerated N sources. With many climate models predicting a shift toward increased stratification and oligotrophy leading toward a major reduction in C flux (Moore et al., 2018), a better understanding of the role of DOM and the organisms involved in its transformations is crucial.

## Author Contributions

MM and DC designed the research and wrote the manuscript. MM conducted the research, analyzed and synthesized the data.

## Conflict of Interest Statement

The authors declare that the research was conducted in the absence of any commercial or financial relationships that could be construed as a potential conflict of interest.
