# Supplementary material for: Direct Utilization of Organic Nitrogen by Phytoplankton and Its Role in Nitrogen Cycling Within the Southern California Bight
Source: Front Microbiol. 2018 Sep 13;9:2118. doi: 10.3389/fmicb.2018.02118 (PMC6146108; doi:10.3389/fmicb.2018.02118)
Supplement: Supplementary file 1 [file Table_1.PDF]

Supplementary Table 1: Characteristics of sampling site, grouped by substrate.

% light	Substrate	Depth (m)	PM (μM N)	std	N uptake (nM h^{-1})	std	N uptake (nM d^{-1})	std	PM turnover (% h^{-1})	ratio of uptake by substrate
50%	UREA	5	1.04	0.3	4.01	0.7	96.3	16.3	0.4	0.3
10%	UREA	17	1.8	0.08	6.2	2.2	149.8	53.3	0.3	0.4
1%	UREA	35	0.9	0.03	2.7	0.2	65.1	5.2	0.3	0.2
50%	NO_3^-	5	1.3	0.5	2.2	0.7	52.0	16.8	0.2	0.1
10%	NO_3^-	17	1.5	0.6	2.3	1.4	56.1	33.1	0.2	0.1
1%	NO_3^-	35	1.4	0.2	8.9	2.2	213.1	53.8	0.6	0.6
50%	NH_4^+	5	1.5	0.7	1.2	0.2	29.0	4.5	0.08	0.1
10%	NH_4^+	17	1.3	0.4	1.4	0.6	34.4	13.2	0.1	0.1
1%	NH_4^+	35	1.2	0.1	7.7	0.8	184.1	18.3	0.6	0.7
50%	N_2	5	1.4	0.6	1.1	0.5	25.8	11.8	0.08	0.8
10%	N_2	17	1.2	0.5	0.26	0.2	6.4	4.2	0.02	0.2
1%	N_2	35	1.2	0.2	0.0	0.0	0.0	0.0	0.0	0.0
