# Supplementary figures and images for: Direct Utilization of Organic Nitrogen by Phytoplankton and Its Role in Nitrogen Cycling Within the Southern California Bight
Source: Front Microbiol. 2018 Sep 13;9:2118. doi: 10.3389/fmicb.2018.02118 (PMC6146108; doi:10.3389/fmicb.2018.02118)

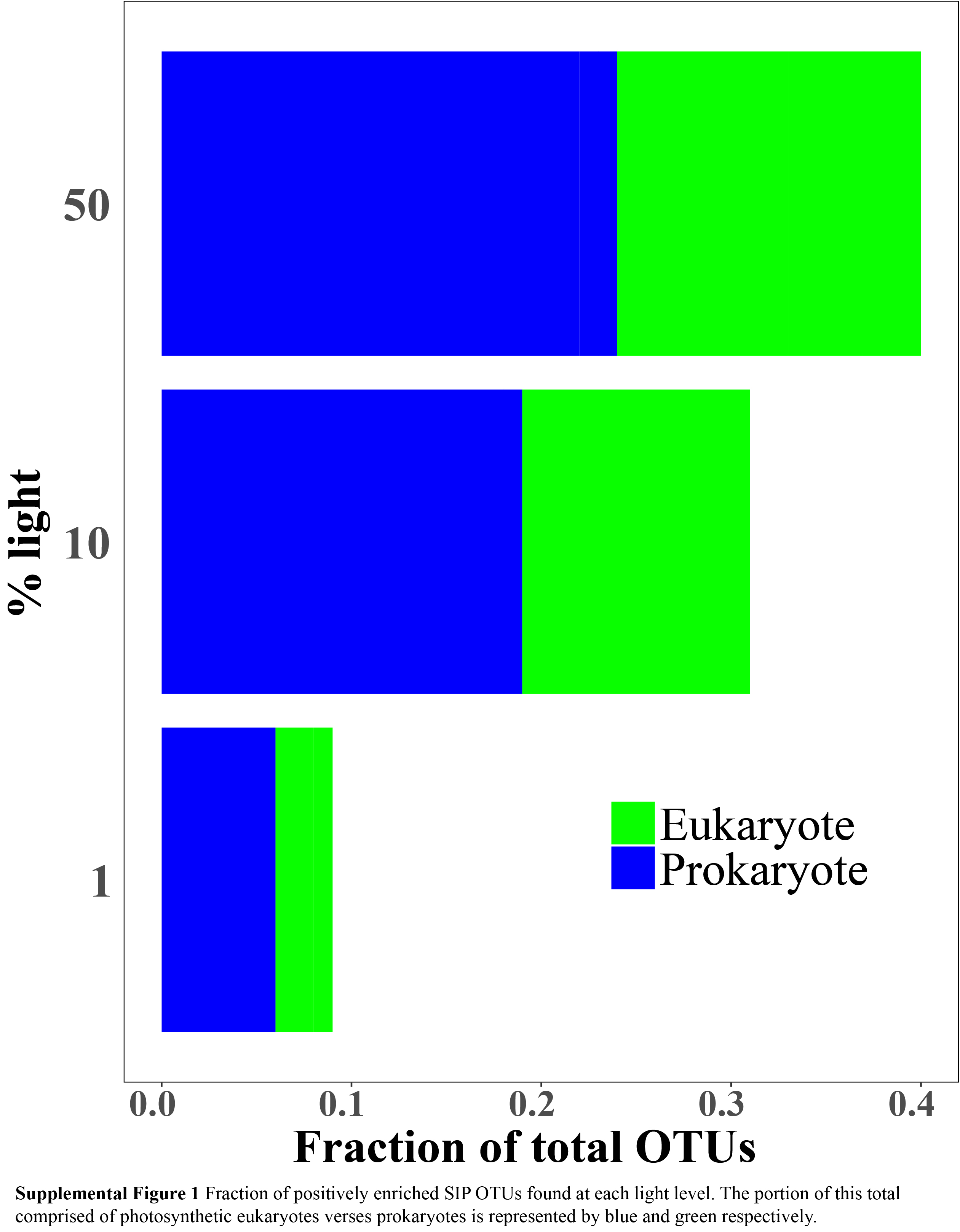

Supplement: Supplementary file 2 [file Image_1.jpeg]

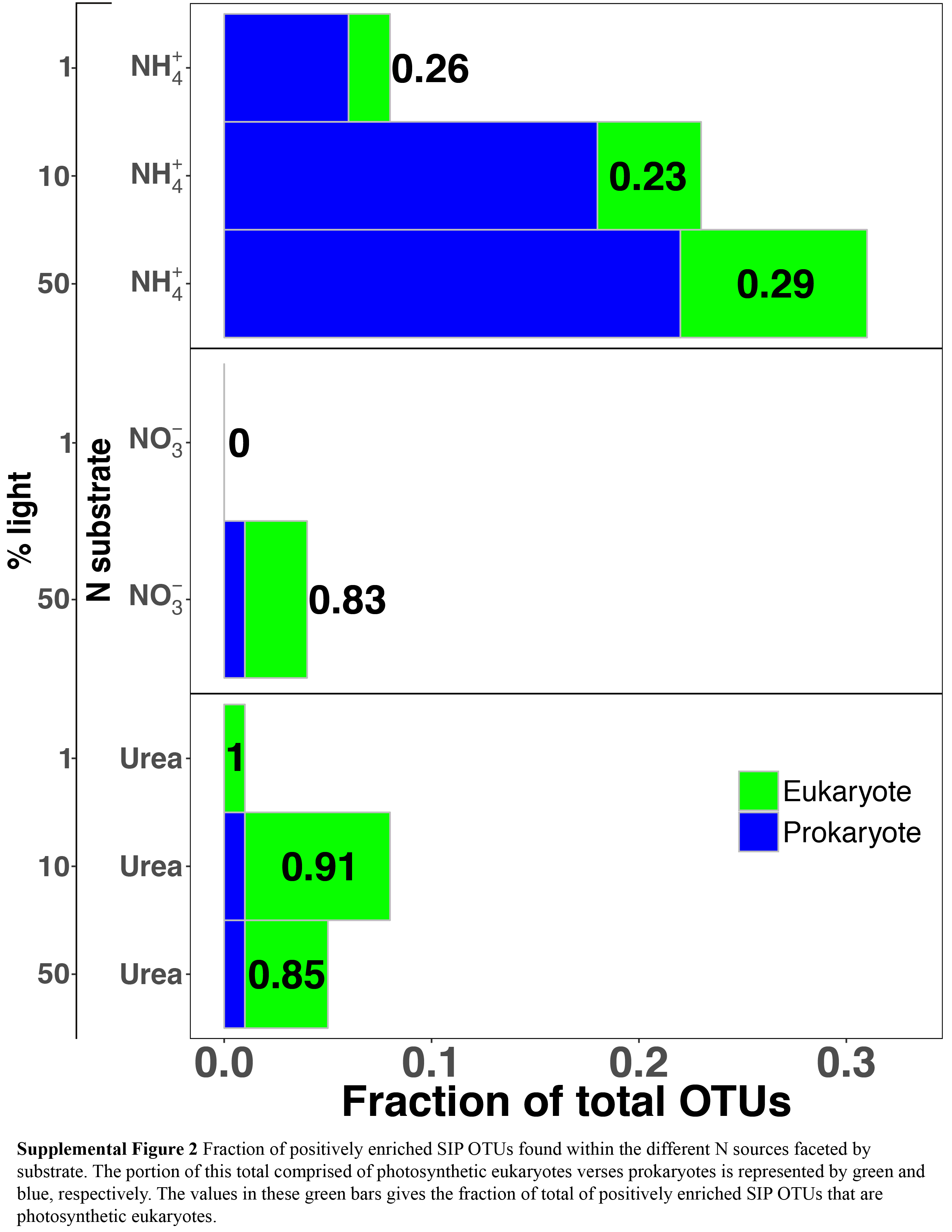

Supplement: Supplementary file 3 [file Image_2.jpeg]

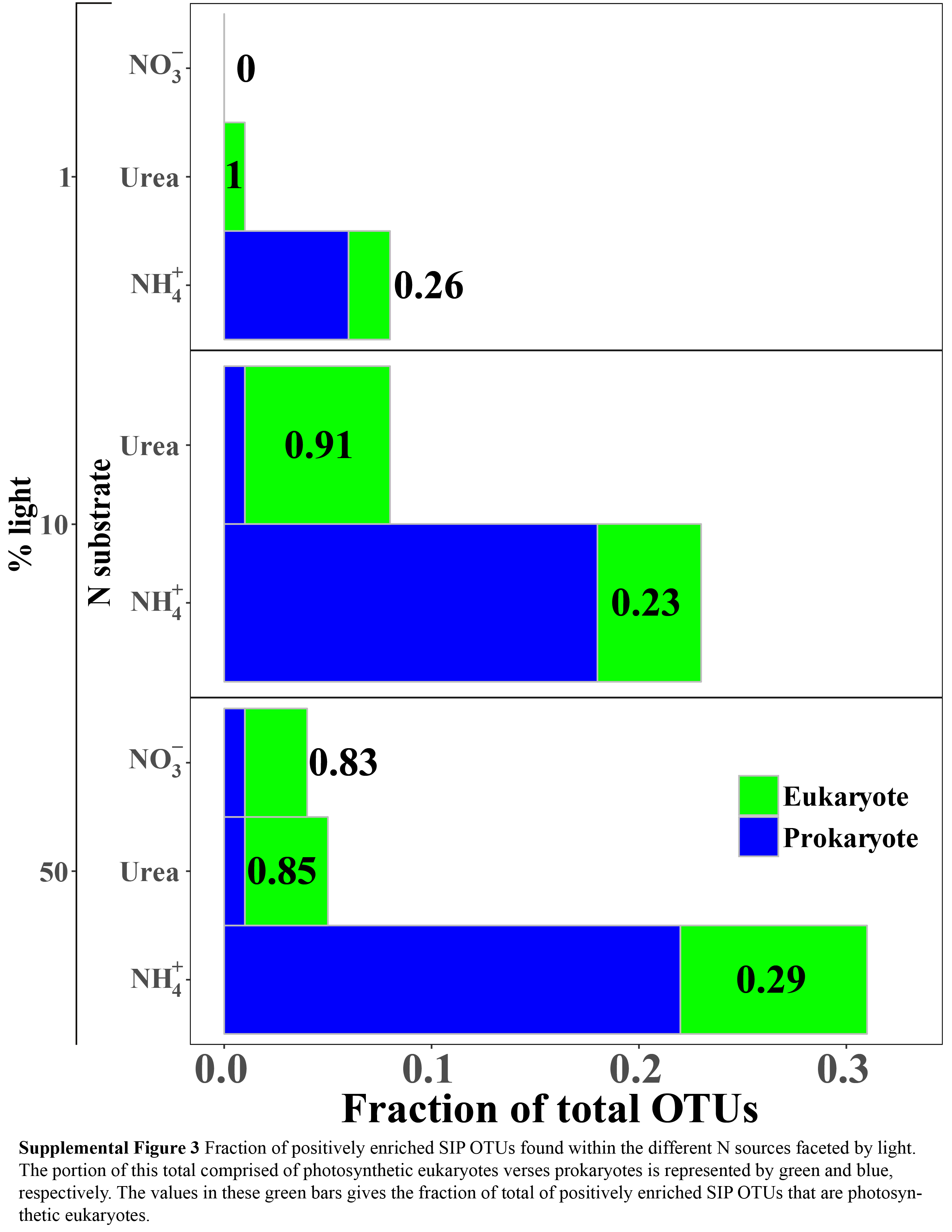

Supplement: Supplementary file 4 [file Image_3.jpeg]

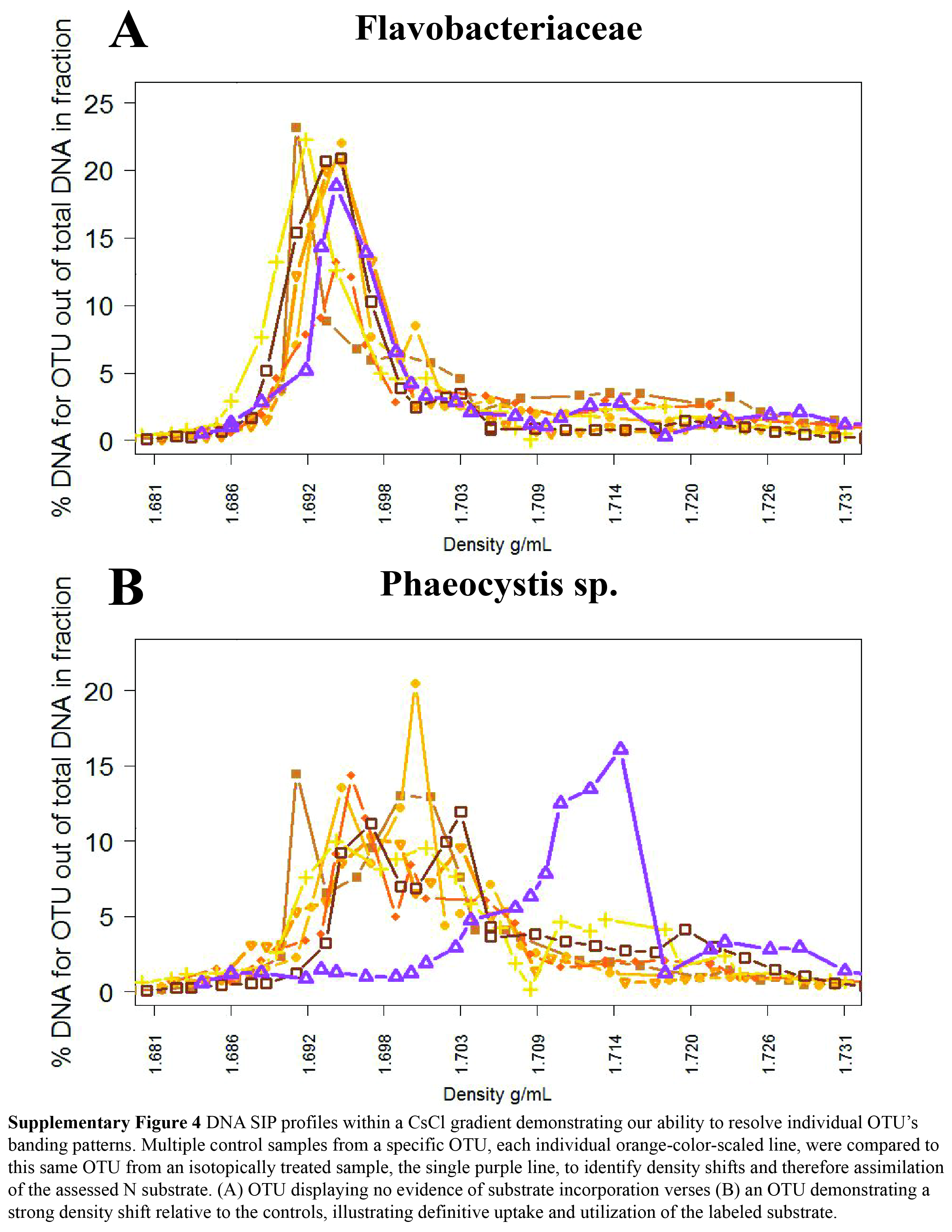

Supplement: Supplementary file 5 [file Image_4.jpeg]
